# Electromagnetic Interference Shielding Anisotropy of Unidirectional CFRP Composites

**DOI:** 10.3390/ma14081907

**Published:** 2021-04-11

**Authors:** Jun Hong, Ping Xu

**Affiliations:** Interdisciplinary Graduate School of Science and Technology, Shinshu University, Ueda 386-8567, Japan; 19hs110e@shinshu-u.ac.jp

**Keywords:** unidirectional CFRP, EMI shielding, anisotropy, free-space measurement

## Abstract

Carbon fiber-reinforced polymer (CFRP) composites have excellent mechanical properties and electromagnetic interference (EMI) shielding performance. Recently, their EMI shielding performance has also attracted great attention in many industrial fields to resolve electromagnetic pollution. The present paper mainly investigated the EMI shielding anisotropy of CFRP materials using a specified set-up of free-space measurement. The electrical conductivity of unidirectional CFRP composites was identified to vary with the fiber orientation angles, and the formula was proposed to predict the results consistent with the experimental. The obvious EMI shielding anisotropy of unidirectional CFRP composites was clarified by free-space measurement. The theoretical formula can predict the EMI shielding value at different carbon fiber orientation angles, and the predicted results were highly consistent with the experimental results. A comparison of the free-space measurement and the coaxial transmission line method was also conducted, which indicated that special attention should be paid to the influence of the anisotropy of CFRP composites on the shielding results. With those results, the mechanism of EMI shielding anisotropy of CFRP composites is clarified, which will provide an effective design of EMI shielding products with a designable shielding direction and frequency.

## 1. Introduction

Recently, with the rapid development in communication and electronic devices, electromagnetic (EM) pollution has become a serious concern that can lead to electromagnetic compatibility (EMC) issues in electronic equipment and the resulting EM radiation of specific frequencies could be harmful to human health [1,2,3,4,5]. This is especially true as the market for 5G technologies and autonomous vehicles are growing rapidly around the world [6,7]. EM radiation usually causes electromagnetic interference (EMI) with other electronics, which can lead to electronic device malfunction [8]. Hence, research focused on effectively solving and avoiding EM pollution has attracted considerable attention.

Nowadays, shielding materials play a key role in protecting electronic equipment from EMI and prevent such radiation sources from emitting radio waves. EMI shielding refers to the absorption or reflection of EM waves with efficient shielding materials, which are composed of either conductive or magnetic-based materials [9,10]. In the past few decades, metal materials have been widely used as EMI shielding materials for excellent shielding performance, but they have disadvantages of low corrosion resistance, poor mechanical flexibility, etc. [11,12].

MXenes have been studied as EMI shielding materials due to their outstanding EMI shielding performance, low density and special metallic features [13,14]. The MXenes composites with different structures have been fabricated to improve its EMI shielding performance [15]. Besides, carbon materials have been investigated as alternatives to metal-based composites to provide great EMI shielding performance. Polymer composites reinforced with carbon-based fillers, such as carbon fibers, graphite, carbon nanotube (CNT), graphene and carbon black have been widely studied for use as shielding materials to provide great shielding abilities to EM waves [16,17,18,19,20,21,22]. Conventional carbon fiber reinforced polymer (CFRP) composites show not only good shielding properties, but also achieve excellent mechanical performance, which can expand their applications in the aeronautic industry [23,24,25]. Other research works [26,27] also observed excellent shielding performance of short carbon fiber reinforced composites, but its mechanical properties and EMI shielding performance were inferior to continuous carbon fiber composite materials [28,29]. Besides, the EMI shielding performance of CFRP could also be improved by being coated with conductive paints filled with metallic particles or nanowires [30], but it still meets the challenge of poor dispersion of conductive fillers and poor wear. In recent years, intrinsically conducting polymers (ICPs) have also been used as fillers for EMI shielding [31,32,33], but their poor mechanical and thermal properties limit their practical applications [34].

The shielding materials should obtain excellent EMI shielding performance in the specified frequency range to meet different practical applications. Many studies have achieved the purpose of improving the EMI shielding performance of composite materials in a specific frequency band by controlling the filler’s size. Jana et al. [35] found that the composite with high carbon fiber aspect ratio (L/D = 100) shows higher shielding effectiveness in the frequency range of 8–12 GHz (X-band). Zhao et al. [36] reported that shielding performance of CFRP was enhanced with the decrease in the array spacing and the increased number of layers in the frequency range of 30–750 MHz, but the number of layers exhibited little effect on the EMI shielding of CFRP at the frequency range of 750 MHz–1.5 GHz. The shielding property of carbon nano-fiber-based composites is also improved in the X-band by increasing the magnetic particle size introduced into the matrix [37]. As above all, shielding materials with various filler sizes and structures exhibit different shielding performances in various frequency bands. In this study, EMI shielding anisotropy of unidirectional CFRP composites was investigated in the frequency range of 5–15 GHz, which is often used in wireless computer networks, radar and satellite communication.

Continuous carbon fiber-reinforced polymer composites have been widely used as structural materials for lightweight structures and EMI shielding applications. However, most researches have focused on the improvement of EMI shielding performance by adjusting the fiber lay-up arrangement or structure [36,38]. Wen et al. [39] reported that the EMI shielding effectiveness (SE) of PVB/Ni-Gr/SCF films exhibited different performances along and perpendicular to the casting direction, where the short carbon fiber direction was changed. Hong et al. [40] indicated that the orientation of fillers could play an important role in determining EMI SE in the polymer-based composites with magnetically responsive aligned Fe_3_O_4_ decorated reduced graphene oxide. The previous works [39,41] revealed that the distribution state of fillers plays an important role in the shielding performance of composites, but the shielding anisotropy was not analyzed further. The carbon fiber orientation has a significant influence on the EMI shielding performance of CFRP composites. However, the EMI shielding anisotropy of unidirectional CFRP, especially its shielding anisotropy mechanisms and SE prediction, has not been systematically investigated and reported. This paper aims to provide a theoretical analysis of the shielding anisotropy of CFRP composites and propose formulas to predict electrical conductivity and EMI SE of CFRP at different orientation angles.

This study mainly discussed the EMI shielding anisotropy of unidirectional CFRP composites. A specially designed free-space measurement was set up in a one direction vibration of the incident EM wave, which can measure the EMI shielding performance in any specified direction. The EMI shielding anisotropy of unidirectional CFRP composites was discussed, and the theoretical formula was used to predict the EMI SE of CFRP at various carbon fiber orientations. The shielding results of the free-space measurement and coaxial transmission line method were compared, and the influence of the EMI shielding anisotropy on the test results with different measurements was discussed.

## 2. Materials and Methods

### 2.1. Fabrication of Unidirectional CFRP Composites

The XN80 carbon fiber-based unidirectional prepregs were supplied by Japan Graphite Fiber Co., Ltd. The carbon fiber areal density was 125 g/m^2^, and the thickness of an individual prepreg was about 0.165 mm. The XN80 carbon fiber had an excellent tensile strength (3430 MPa), tensile modulus (780 GPa), electrical conductivity (5 × 10^−4^ ohm cm), and the fiber diameter was about 10 μm. A four-layered unidirectional CFRP composite was made of four single prepregs arranged in the same fiber direction. The CFRP composites were cured by the hot press machine at 135 °C and 2 MPa for 1.5 h. The CFRP composite was a square with a side length of 25 cm and a thickness of 0.55 mm.

### 2.2. Electrical Conductivity of CFRP Composites

The electrical conductivity of the unidirectional CFRP composites was tested by the four-probe method [28]. All specimens had a uniform dimension of 80 mm × 10 mm × 0.55 mm, and a silver paint was used to ensure good contact between the electrodes and specimens during testing. Four electrical contacts were symmetrically positioned relative to the center of the specimen, and the distance between the adjacent electrical contact was about 20 mm. Each electrical contact with silver paint was 2 mm wide. A current of 0.1 mA was provided by the DC power supply device (Takasago EX-375L2, Takasago Ltd., Kanagawa, Japan) at the outer electrical contacts, and the digital electrometer (Advantest R8240, Advantest Corporation, Tokyo, Japan) was used to measure the voltage at the inner contacts. Each test was carried out 5 times. Since the carbon fibers were arranged in the same direction in the unidirectional CFRPs, their ability to transfer electrons was different at various fiber orientations. The electrical conductivity of the specimens was measured at 0°, 15° 30°, 45°, 60°, 75°, and 90°. Where the 0° direction refers to the fiber direction, and 90° refers to the direction perpendicular to the carbon fiber.

### 2.3. EMI Shielding Measurement

**Figure 1:**
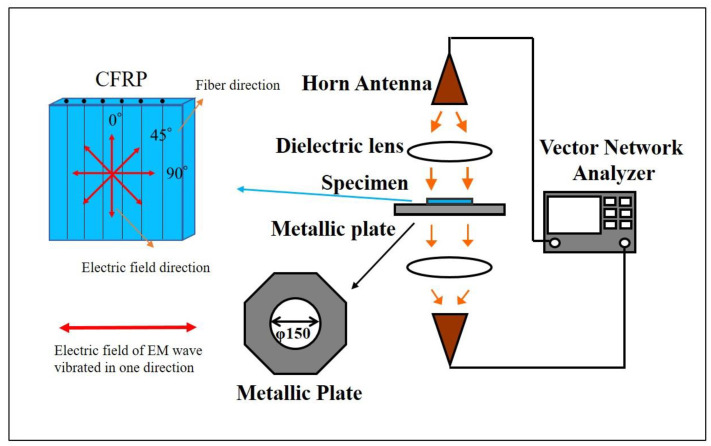
Schematic of free-space set-up for electromagnetic interference (EMI) shielding measurement. CFRP: carbon fiber-reinforced polymer.

**Figure 2:**
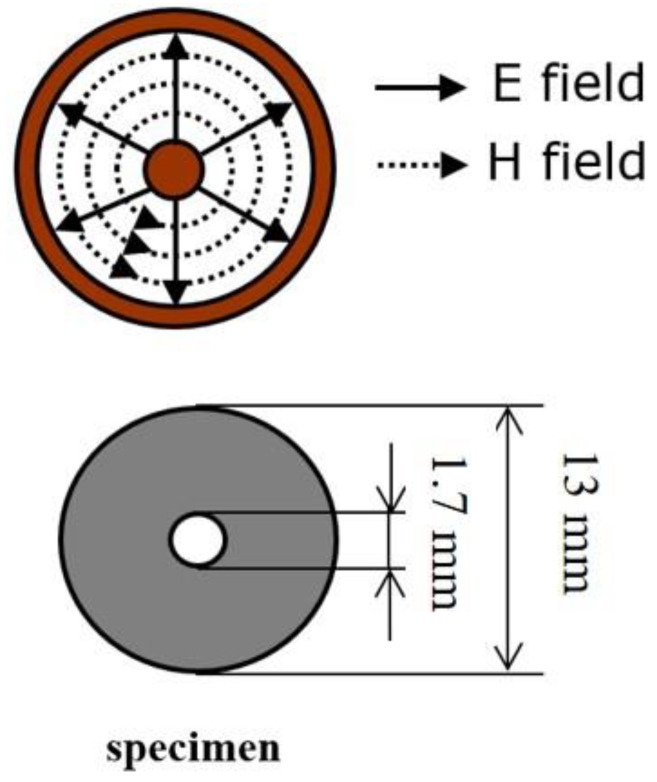
Distribution of the electric field inside the coaxial tube and the specimen dimension used for coaxial transmission line measurement.

The EMI shielding performance of the CFRP composites was test by the free-space measurement method. The specially designed free-space measurement system (KEYCOM RTS03, KEYCOM Corporation, Tokyo, Japan) consists of two antennas, two lens and a sample stage, as shown in Figure 1. The electric field of the incident EM waves was linearly polarized. Figure 2 shows the distribution of the electric field inside the coaxial tube and the specimen dimension used for coaxial transmission line measurement (KEYCOM S-GPC7, KEYCOM Corporation, Tokyo, Japan). The value of the scattering parameters (*S*_21_ or *S*_12_) of the CFRP materials were tested by the vector network analyzer (Anritsu 37247D, Anritsu Corporation, Kanagawa, Japan). The attenuation of the EM wave was defined as the *SE*, which can be calculated by the following equations:

(1) $$\;\;\;\;SE=10\log\left(\frac{P_{T}}{P_{I}}\right)$$

(2) $$SE=-10\log\left({\left|S_{21}\right|}^{2}\right)=-10\log\left({\left|S_{12}\right|}^{2}\right)$$

where *P_T_* is the transmitted power, *P_I_* is the incident power, and the *S*_12_ *(S*_21_*)* parameters refer to the transmission coefficients.

## 3. Results

### 3.1. Electrical Conductivity of Unidirectional CFRP Composite

**Figure 3:**
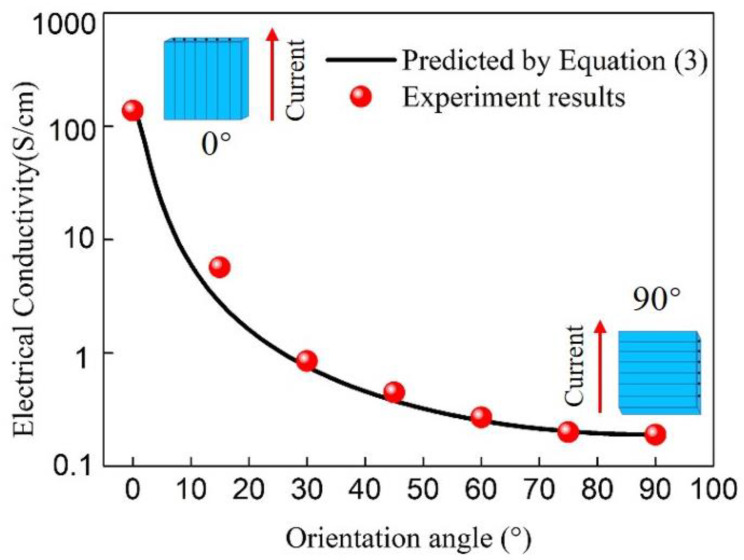
Electrical conductivity of the CFRP at different carbon fiber orientation angles.

Figure 3 shows the electrical conductivity of CFRP at the different fiber orientations of 0°, 15°, 30°, 45°, 60°, 75°and 90° (red dots). The conductivity of CFRP decreased as the fiber orientation angle increased from 0° to 90°. An anisotropic CFRP composite was described as that all the fibers are highly aligned and parallel to each other in the composite. However, the fibers are not entirely straight and have a wave shape in the composite, consequently creating many contact points between adjacent fibers. This causes the composite to have specific electrical conductivity in the transverse direction of the fiber, although it is extremely lower compared with that in the fiber direction. As a consequence, the unidirectional CFRP composite shows the highest and lowest conductivity values in the fiber direction and transverse direction, respectively.

The decreasing tendency of conductivity was not linear of CFRP, which decreased as the fiber orientation angle increased from 0° to 90°. The conductivity decreased sharply from 0° to around 30°, and the decreasing trend became gradual when exceeding 30°. Since the conductivity of CFRP presents a cosine change trend, the conductivity at different angles can be calculated and predicted by the formula derived below:

(3) $$\sigma_{\theta }=\frac{1}{A\cos^{2}(\theta +\pi )+B}\;\;0\leq \theta \leq \frac{\pi }{2}$$

where *σ_θ_* is the electrical conductivity of CFRP at *θ* degree, *A* = 1/*σ*_0_ − 1/*σ*_90_, *B* = 1/*σ*_90_, *σ*_0_ and *σ*_90_ are the electrical conductivity of CFRP at the testing angle of 0° and 90°, respectively.

The curve in Figure 3 shows the predicted electrical conductivity of unidirectional CFRP as a function of the carbon fiber orientation angle from 0° to 90°. It can be seen from Figure 3 that the calculated results are in good agreement with the experimental results, indicating that the proposed formula can predict the electrical conductivity of unidirectional CFRP at any angle just through the conductivity values at 0° and 90° directions.

### 3.2. Skin Depth of Unidirectional CFRP Composite

When the EM wave is incident on the surface of the material, the displacement current of the wave is coupled to it and generates the magnetic field at a right angle, which can create a back electromotive force to cause a force named the “skin effect” [42]. As the propagation depth increase, more EM waves are attenuated. When the strength of the EM wave is reduced to 1/e of the incident strength, the distance is known as the skin depth [43], and can be calculated by the following equation [44]:

(4) $$\delta =1/\sqrt{\pi \mu f\sigma }$$

where *δ* is the skin depth, *f* is the frequency, and *σ* is the electrical conductivity of the material. The magnetic permeability *μ = μ*_0_*μ_r_*, where the magnetic permeability of vacuum (*μ*_0_) is 4π × 10^−7^ H/m, and the relative magnetic permeability (*μ_r_*) of CFRP is about 1 [28,45].

**Figure 4:**
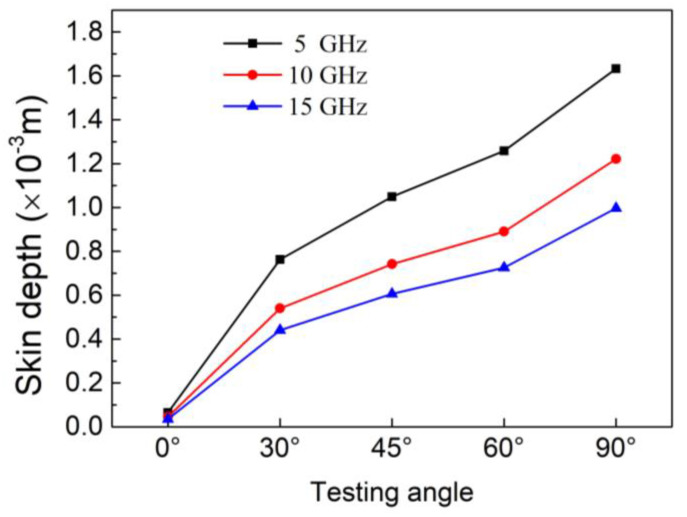
Skin depth of CFRP as a function of testing angles.

Figure 4 shows the skin depth of unidirectional CFRP as a function of carbon fiber orientation angles at different frequencies. The skin depth of the composite decreased with an increase in the orientation angle. This is mainly due to the decrease in conductivity as the carbon fiber orientation angle increases, as shown in the conductivity results in Figure 3. The skin depth also decreases as the frequency increases. At a frequency of 15 GHz, the CFRP had the lowest skin depth value of 0.035 mm in the 0° direction, which refers to the fiber direction. At 5 GHz, CFRP obtained a maximum value of 1.633 mm in 90° direction.

The thickness of the fabricated unidirectional CFRP was about 0.55 mm. According to the definition of skin depth, when the frequency is 15 GHz, and the electric field of the EM wave is consistent with the fiber direction, the electric field intensity would be less than 1/e of the incident electric field intensity when the EM waves penetrate through the CFRP composite. At 5 GHz, when the electric field is perpendicular to the fiber direction, the electric field intensity would be greater than 1/e of the incident electric field intensity when the EM waves pass through the CFRP. Consequently, the skin depth of materials plays a key role in the attenuation of EM waves, and due to the dependence of the skin depth on the carbon fiber orientation angle, the EMI shielding performance of CFRP would be different at various carbon fiber directions.

### 3.3. EMI Shielding Theory of CFRP Composite

**Figure 5:**
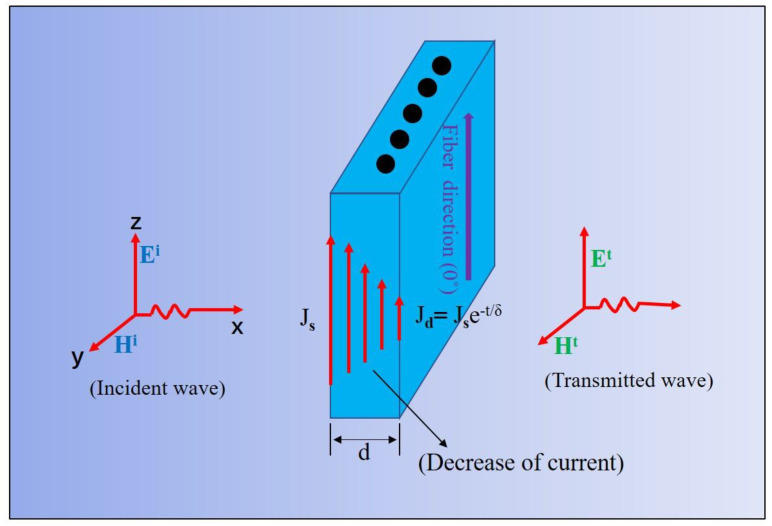
Schematic of EMI shielding mechanism of unidirectional CFRP.

According to the circuit theory [42] for calculating the EM wave attenuation by shielding, the displacement current in the wave would be coupled to the CFRP when the EM wave encounters the CFRP composite, as shown in Figure 5. Then, the surface current density onto the CFRP will generate a magnetic field (H) perpendicular to it, and the magnetic field would create an opposite electromotive force, which can attenuate the current to penetrate the CFRP. This phenomenon leads to the attenuation of EM waves. The shielding attenuation *S(dB)* for the electric field (E) can be obtained as follows:

(5) $$\;S\left(dB\right)=20\log\frac{E_{I}}{E_{T}}\;\;=20\log\frac{J_{I}Z_{W}}{J_{T}Z_{C}}$$

where *E_I_* and E_T_ are the electric field at the incident side and transmitted side, respectively; *J_I_* is the surface current density at the incident side of the CFRP, and the current value at the transmitted side *J_T_* is expressed as:

(6) $$J_{T}=J_{I}e^{-d/\delta }\;$$

where *d* is the thickness of CFRP, and *δ* is the skin depth.

*Z_W_* and *Z_C_* are the impedance of the incident wave and CFRP composites, respectively, which is equal to:

(7) $$Z_{W}=-\frac{j377\lambda }{2\pi L}\;\left(L<\frac{\lambda }{2\pi }\right)\;=377\;\;\left(L\geq \frac{\lambda }{2\pi }\right)$$

(8) $$\;Z_{C}=\frac{1+j}{\sigma \delta \left(1-e^{-d/\delta }\right)}\;$$

where *σ* is the electrical conductivity of the CFRP composite. Then, the shielding attenuation *SE (dB)* of the CFRP composite can be expressed as:

(9) $$SE\left(dB\right)=20\log\frac{Z_{W}}{e^{-d/\delta }\cdot Z_{C}}.$$

**Figure 6:**
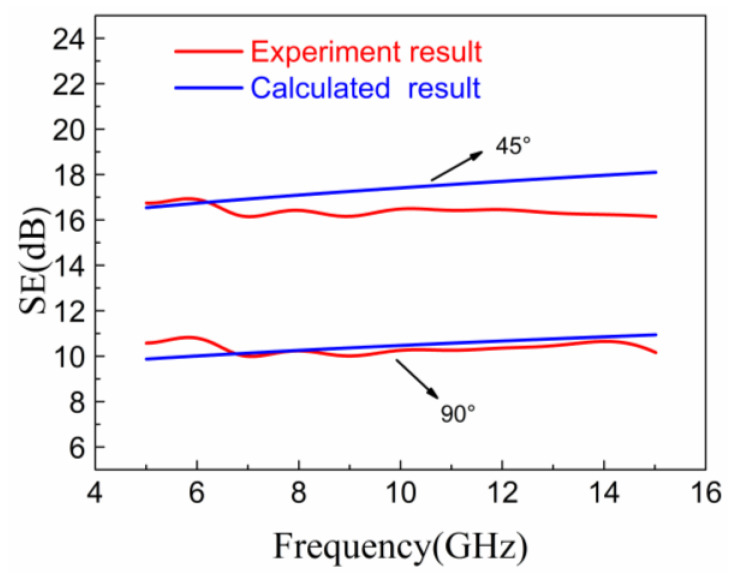
Comparison of experimental value and formula calculation value of EMI SE.

According to Equation (9), the thickness and skin depth of the material plays a decisive role in the EM shielding performance. The formula’s applicability in the frequency range of 5–15 GHz was verified by taking the test angles of 45° and 90° as examples. The skin depth, electrical conductivity, and related parameters were substituted into Equation (9). The comparison between the calculated prediction and the experimental results by the free space measurement is shown in Figure 6. It was observed that the experimental and predicted result were in good agreement. The maximum difference at fiber orientation of 45° is only about 1.9 dB. It can be concluded that Equation (9) can be applied to predict the EMI SE of unidirectional CFRP at the frequency range of 5 to 15 GHz.

**Figure 7:**
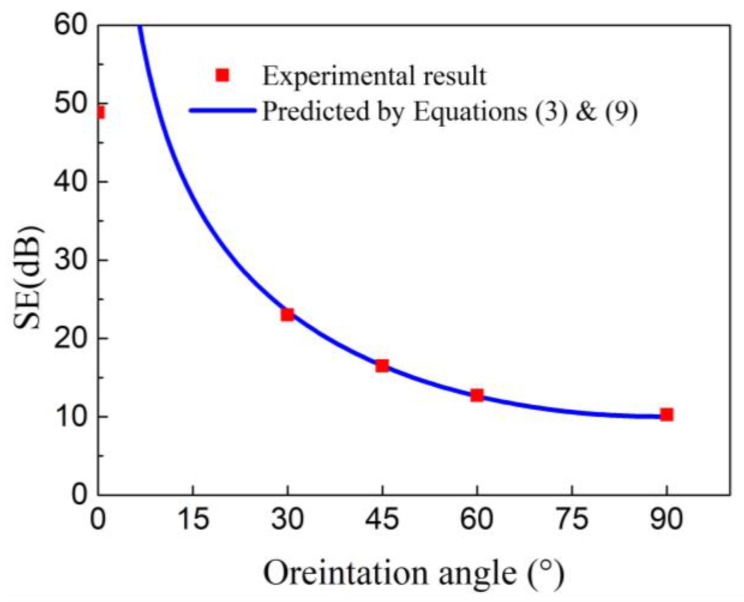
EMI SE of CFRP for experimental and predicted results at the frequency of 10 GHz.

By combining Equations (3) and (9), the EMI SE of unidirectional CFRP could be predicted at any carbon fiber orientation angles. Figure 7 shows that the predicted results are highly consistent with the experimental results in the various fiber directions at 10 GHz. When only using Equation (9) to calculate the shielding performance of CFRP at different orientation angles, it is necessary to test the material’s EM parameters (skin depth, electrical conductivity, etc.) in each direction. Here, Equations (3) and (9) are used in combination, and it is only necessary to calculate or measure the electromagnetic parameters of CFRP in the 0° and 90° directions and substituting the results into the Equation (9) to obtain the EMI SE at any carbon fiber orientation angles. The predicted results were highly in good agreement with the experimental values. There was a difference between the experimental and calculated results in the fiber direction (0°). That might be due to the device limitation; that is, the EMI SE of the CFRP materials might have exceeded the measuring range of the device in the fiber direction.

### 3.4. Comparison of Coaxial Transmission Line Method and Free-Space Measurement

**Figure 8:**
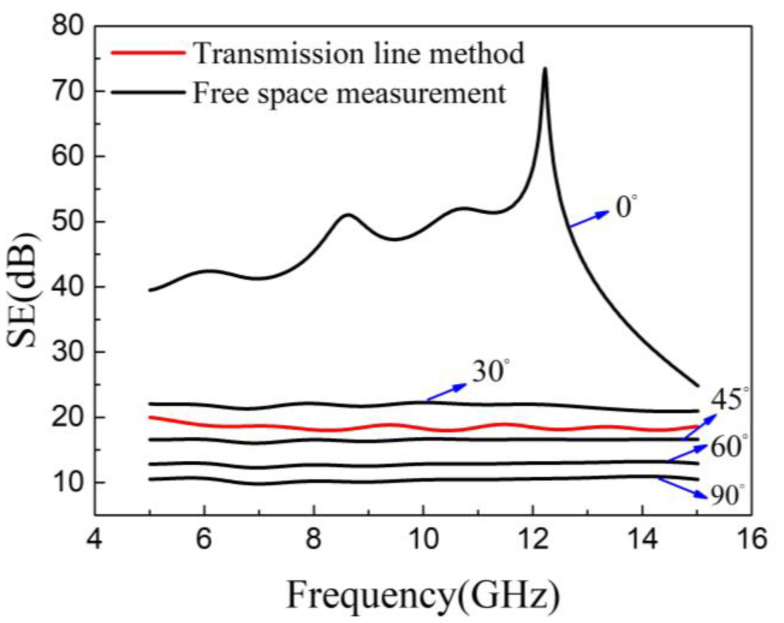
EMI SE of CFRP by the transmission line method and free-space method.

To further understand the relationship between the EMI shielding performance and carbon fiber orientation, the shielding result obtained by the transmission line method was compared to that by free-space measurement. The EMI shielding performance of unidirectional CFRP with a carbon fiber orientation angle of 0, 30, 45, 60, and 90° at the frequencies from 5 to 15 GHz are shown in Figure 8. CFRP showed an excellent electromagnetic shielding value when the carbon fiber orientation angle is 0°, where the electric field polarization direction of EM waves is parallel to the fiber direction. The SE of CFRP decreased as the fiber orientation angle increased due to the decrease in electrical conductivity. When the orientation angle was 90°, the electric field polarization direction was perpendicular to the carbon fiber, and the CFRP obtained the lowest electrical conductivity in this orientation angle. Thus, CFRP showed the lowest SE value of about 10 dB.

The red curve in Figure 8 represents the EMI shielding result measured by using the coaxial transmission line method. The SE value obtained by this method was about 19 dB. This is due to the fact that the electric field of EM waves was quasi-radially distributed inside the coaxial tube (Figure 2), which has been discussed in our previous work [46]. When the coaxial transmission line method was adopted to evaluate the EMI shielding performance of unidirectional CFRP, the EMI shielding result did not change if the position of the composite material was rotated along the inner conductor. However, under the free-space measurement system, the EMI shielding performance greatly depended on the test angle. Unidirectional CFRP exhibits remarkable EMI shielding anisotropy. This characteristic of the one-side composite material can be used for the bias material of EM wave radiation, meaning that the EM waves polarized in a specific direction can be selectively shielded by adjusting the angle of the CFRP composites.

The results show that when different test methods are used to evaluate the shielding performance of unidirectional CFRP composites, the results will differ. Therefore, when assessing anisotropic materials, especially materials prepared by CFRP prepregs, special attention should be paid to the influence of electromagnetic shielding anisotropy.

## 4. Conclusions

Carbon fiber-reinforced composite materials occupy a critical position in many structural material applications, and their EM shielding performance has also attracted great attention. This study mainly investigated the EMI shielding anisotropy of CFRP materials.

The electrical conductivity of unidirectional CFRP composites varies due to the fiber orientation angles, and the calculated results by the formulas proposed in this study were highly consistent with the experiment values. It was found that the skin depth of unidirectional CFRP was different at various orientation angles and frequencies. At a frequency of 15 GHz, the CFRP exhibited the lowest skin depth value of 0.035 mm in the 0° direction. All the characteristics mentioned above are key issues leading to the EMI shielding anisotropy of CFRP composites. The obvious EMI shielding anisotropy of unidirectional CFRP composites is clarified by the experimental results using the specified set-up of free-space measurement. The shielding results predicted by the theoretical formula are highly consistent with the experimental results. The maximum difference between the predicted and experimental values at a fiber orientation of 45° is only about 1.9 dB.

A comparison of the free-space measurement and the coaxial transmission line method was also conducted, and the influence of electric anisotropy on the test results was further discussed. The SE values obtained by the coaxial transmission line method was much lower than that obtained by the free-space measurement tested in the fiber direction, which indicated that special attention should be paid to the influence of the anisotropy of CFRP composites on the shielding results when evaluated by various measurements. With these results, the mechanism of EMI shielding anisotropy of CFRP composites is clarified, which will provide an effective design of EMI shielding products with a designable shielding direction and frequency.
